# New human rabies vaccines in the pipeline

**DOI:** 10.1016/j.vaccine.2018.08.039

**Published:** 2019-10-03

**Authors:** Anthony R. Fooks, Ashley C. Banyard, Hildegund C.J. Ertl

**Affiliations:** a Animal and Plant Health Agency, Weybridge, Surrey, UK; b Wistar Institute, Philadelphia, PA, USA

**Keywords:** Pre-exposure prophylaxis (or PrEP), Post-exposure prophylaxis (PEP), Rabies immune globulin (RIG), Correlates of protection

## Abstract

Rabies remains endemic in more than 150 countries. In 99% of human cases, rabies virus is transmitted by dogs. The disease, which is nearly always fatal, is preventable by vaccines given either before and/or after exposure to a rabid animal. Numerous factors including the high cost of vaccines, the relative complexity of post-exposure vaccination protocols requiring multiple doses of vaccine, which in cases of severe exposure have to be combined with a rabies immune globulin, lack of access to health care, and insufficient surveillance contribute to the estimated 59,000 human deaths caused by rabies each year. New, less expensive and more immunogenic rabies vaccines are needed together with improved surveillance and dog rabies control to reduce the death toll of human rabies. Here, we discuss new rabies vaccines that are in clinical and pre-clinical testing and evaluate their potential to replace current vaccines.

## 1 Introduction

Although vaccines that can prevent a rabies virus infection are commercially available, the death rate due to rabies encephalitis has more or less remained steady for decades. Human rabies occurs in 150 countries around the globe causing about 59,000 human deaths each year; although due to underreporting from developing countries the actual number is thought to be far higher [1]. Most cases are reported from Africa and Asia and ∼40% of deaths affect children under the age of 14. Rabies is most commonly (more than 99% of cases) transmitted by dogs and although other mammals also transmit rabies virus; they account for less than 1% of human rabies cases.

Rabies virus is a member of the Lyssavirus genus. Lyssaviruses, all 16 of which are capable of causing rabies, are generally divided into two major phylogroups. Phylogroup I includes Rabies lyssavirus, Aravan lyssavirus, Australian bat lyssavirus, Bokeloh lyssavirus, Duvenhage lyssavirus, European bat-1 and bat-2 lyssaviruses, Irkut lyssavirus, Khujand lyssavirus, and Gannoruwa bat lyssavirus, while Lagos bat lyssavirus, Mokola lyssavirus, and Shimoni bat lyssavirus belong to phylogroup II [2]. Three further lyssaviruses, Ikoma lyssavirus, West Caucasian bat virus, and Lleida bat lyssavirus have also been characterized that fall outside of these two defined phylogroups. The majority of these viruses have been identified in bats and bats have been proposed as the host reservoir for all lyssaviruses with only Rabies lyssavirus commonly circulating in terrestrial carnivores. Existing vaccines are based on Rabies lyssavirus and protect against phylogroup I lyssaviruses but limited antigenic cross-reactivity between phylogroup I and II viruses precludes reliable protection against the latter [3], [4].

Rabies, once symptomatic, is fatal in 99.9% of human cases and most of the human survivors have retained significant neurological sequalae. Rabies virus once transmitted by the saliva of an infected animal or through contact with infected tissues enters peripheral nerve endings and through axonal transport reaches the central nervous system (CNS). In the CNS, the virus replicates and then travels back to highly innervated tissues such as salivary glands from where it can be transmitted to the next victim. The incubation time for human rabies is highly variable. On average initial symptoms such as discomfort at the infection site, fever or general weakness commence 3–6 weeks after exposure [5] and then rapidly progress to a full-blown infection that can either take a furious or paralytic form. Patients with furious rabies exhibit symptoms of anxiety, confusion, fear of water due to problems swallowing, agitations and hallucinations while paralytic rabies patients progress from an ascending motor weakness to a coma [6]. In approximmately 1–3% of rabies cases the incubation time exceeds 6 months and can even extend over several years [7], [8].

Treatment of symptomatic rabies has been attempted and the so-called Milwaukee protocol [9], which involves intensive therapy during which patients are shielded from neurological stimulations by coma-inducing drugs, and has reported limited success in a few patients. In most patients this protocol [10] and other experimental treatments such as anti-viral drugs, high doses of interferons [11], [12] or transfer of neutralizing antibodies [13] may prolong disease but do not avert death.

Disease can be prevented by vaccines. Rabies vaccines have been available for over a century since the pioneering work of Pasteur and Roux in 1885 established that immunization with desiccated nerve tissue from an infected rabbit was able to prevent disease in a 9-year old boy, who had been attacked by a rabid dog. Since this time, safe and efficacious rabies vaccines have been developed. Despite this, thousands of humans are estimated to still die each year from this dreaded disease with the reasons behind this dichotomy being largely socioeconomic [14]. The majority of humans at risk do not receive PEP after contact with a rabid animal or they receive PEP without RIG. Rabies vaccines and RIG are expensive, and the former have to be given repeatedly. Patients or their parents not only have to carry the costs for the biologicals, medical supplies and treatment but also encounter expenses due to repeated travel to health care facilities and loss of wages. Lack of knowledge of the disease and potential post exposure actions, lack of surveillance in animals and social instability further contribute to absence of essential treatment.

In individuals at high risk for rabies virus exposure the vaccines, which are based on inactivated purified viruses, are given in a pre-immunization (PrEP) regimen. Rabies vaccines are not overly immunogenic compared to live attenuated vaccines and contain no adjuvant so that 3 doses given are needed to reliably induce protective titers of neutralizing antibodies. Different regimens have been approved by the World Health Organization (WHO) [15] such as one-week (days 0, 3 and 7) two-site intradermal, two-week, one-site intramuscular (days 0, 3, 7 and between days 14–28) and 3 week-intramuscular (days 0 [2 sites], 7 [one site] and 21 [1 site] vaccinations. Immunological memory is long-lasting [16], [17] so that upon rabies virus exposure of an already vaccinated individual, one or two doses of vaccine are administered that according to WHO should be given at one site intradermally on days 0 and 3, four sites intradermally only on day 0 or one site intramuscularly on days 0 and 3 [15]. In an unvaccinated individual upon contact with a rabid animal, the severity of exposure dictates if the vaccine has to be combined with a rabies immune serum (RIG). In cases of licks or minor scratches, three regimens (5-and 4-dose regimen, 2-1-1 regimen) are approved for intramuscular post-exposure prophylaxis (PEP) and a 2 site 4-dose intradermal regimen has been adapted by some countries. In cases of more invasive exposures, such as bites, licks of mucosal membranes or exposure to bats, the vaccine needs to be combined with RIG; and most of the RIG should be infiltrated into the wound to prevent further spread of the virus. In either case wounds need to be cleaned thoroughly to neutralize as many live virions as possible [15].

## 2 Correlates of protection

Correlates of protection [18], [19] against rabies virus are well defined. Neutralizing antibodies directed against the rabies virus glycoprotein [20], the only surface protein of this bullet-shaped virus, prevent disease. In general titers of or above 0.5 international units/ml are evidence of seroconversion and considered protective [21] but this number should be viewed with caution [22]. Rabies causing lyssaviruses are a diverse group of viruses. Higher titers, which are typically determined by tests based on Rabies virus, may be needed to reliably protect against lyssaviruses that are antigenically more distant from the vaccine strain. Induction of an immunoglobulin class switched affinity-matured B cell response [23], [24] requires help from CD4^+^ T cells. Although this has not yet been studied for rabies vaccines, B cell help is provided best by follicular T help cells, which in germinal centers within lymphatic tissues provide important cues for differentiation of naïve B cells into long-lived antibody secreting cells and memory B cells [25]. Individuals with inherited or acquired B or CD4^+^ T cell immunodeficiencies will not respond well to a rabies vaccine [26], [27]. In these patients, the use of RIG is mandatory after exposure, and titers should be checked after vaccination and if they remain below 0.5 IU/ml additional doses of vaccine should be given [28]. As testing of antibody titers is costly, this protocol is rarely followed in resource poor-countries.

## 3 Current rabies vaccines

Early rabies vaccines were modified versions of the vaccine that was used in 1885 by Louis Pasteur. They were produced in nerve tissue and then inactivated with phenol. For PEP, 14-21 daily doses were needed to induce adequate titers of antibodies. The potency of these so-called Semple vaccines was highly variable. As Semple vaccines contained residual nerve tissue neurological complications such as neuropathy, Guillain-Barré syndrome, meningitis or encephalitis were common. Nerve tissue rabies vaccines (NTVs) including the Semple and the Fuenzalida rabies vaccine, the latter being grown in suckling mouse brain and thus containing less myelin, are still used in some developing countries [29]. However, NTVs are no longer recommended by the WHO, which endorses the use of vaccines grown in cell culture or embryonated eggs. For WHO approved vaccines rabies virus grown in embryonated duck or chicken eggs, primary chick embryo cells, Vero cells or human diploid cells is upon harvest concentrated, purified, inactivated and lyophilized. Potency is tested, and the vaccine is required to contain equal or above 2.5 international units per intramuscular dose [14].

## 4 Future human rabies vaccines

Next generation human vaccines are in general developed to reduce adverse events or improve efficacy of available vaccines. While early nerve tissue rabies vaccines were highly reactogenic, current vaccines have an excellent safety profile and severe adverse events are scarce. Available rabies vaccines are highly efficacious and upon their appropriate use vaccine failures have been reported [30] but are very rare. Economic factors, rather than concerns about safety or lack of efficacy, are driving the development of new rabies vaccines. Reduced cost and, preferentially, single immunization regimens would be especially valuable to expand pre-exposure prophylaxis (PrEP) and allow for its incorporation into childhood immunization programs in highly endemic areas. The effectiveness of more wide-spread PrEP has been validated in Peru [31]. This country has successfully stopped the increasing incidence of vampire bat-transmitted rabies in children in Amazonia by incorporating the rabies vaccine into routine childhood immunization programs [31]. Nevertheless, the use of current rabies vaccines for childhood PrEP is not cost-effective [32], which necessitates the development of cheaper and more immunogenic single dose vaccines that are stable and do not require cold-chain storage and can be preferentially administered orally or needle-free [33].

The same rabies vaccines are used for both PrEP and PEP. PrEP aims to induce sustained titers of rabies virus neutralizing antibodies and immunological memory. The goal of PEP is to stimulate a rapid neutralizing antibody response following potential exposure. The initial short-lived antibody response composed of IgM develops outside germinal centers and is independent of T cell help. Neutralizing IgM antibodies appear within a few days and, as studies in mice have shown, may contribute to protection [34]. Induction of sustained affinity-matured IgG neutralizing antibody responses and memory B cells takes longer but is essential for PrEP. One could thus argue that different vaccines should be developed to fulfill the needs of PrEP versus PEP. Subunit vaccines based on genetic sequences that encode the rabies virus glycoprotein can depend on the delivery vehicle to induce a potent and sustained antibody and memory B cell responses after a single dose [35]. It takes time for genetic vaccines to first enter cells, become transcribed and translated and produce enough protein to induce an immune response. Genetic vaccines although highly suited for PrEP should thus not be developed for PEP. Adjuvanted rabies vaccines, vaccines based on genetically modified rabies virus or protein vaccines on the other hand are appropriate for PEP.

## 5 Protein vaccines

The rabies virus glycoprotein assembles as a homotrimeric protein on the surface of the virion and most of the antigenic sites are conformational epitopes that depend on the correct quaternary protein structure [36]. Generating cost-effective protein-based vaccines with the correct structure remains a challenge, especially when a crystal-structure for lyssavirus glycoprotein remains undefined. However, a number of expression systems have been explored. Mammalian expression systems based on human embryonic kidney (HEK) 293 [37], baby hamster kidney (BHK)-21 [38], or Chinese hamster ovary (CHO) [39] cell lines have been tested with varied success; they showed distinct patterns of glycosylation depending on cell substrate and culture conditions [40]. Insect cell expression system based on recombinant baculovirus [41] or transfected Drosophila melanogaster Schneider 2 cells [42] tend to be more efficient for high yield protein expression but they typically add shorter glycans with added fucose and xylose residues. Yeast expression system are also cost-effective by their glycosylation with mannose only containing glycans is very different from that of mammalian cells and accordingly yeast-derived rabies virus glycoprotein is poorly immunogenic [43]. The rabies virus glycoprotein has also been expressed by plants such as tomatoes [44], carrots [45], maize [46] and others with the intention to eventually develop edible rabies vaccines. Producing foreign proteins in plants is cost-effective but hampered by purification issues. The latter would not affect edible vaccines but immune responses elicited by oral immunization with plant-derived rabies virus glycoprotein have thus far been variable and in general below those needed to achieve reliable protection [47]. One protein nanoparticle vaccine generated by CPL Biologicals, Gujarat, India, is currently in phase III testing in healthy volunteers in India (https://biospectrumasia.com/article/pdf/9246). This vaccine is based on a recombinant baculovirus-derived glycoprotein that was modified to self-assemble into nanoparticles. Information on the performance of the vaccine in Phase I/II trials is not available. Although protein vaccines are safe they may not be cost effective. They would require extensive purification and addition of an adjuvant. Experiments in pre-clinical models showed that they would not be sufficiently immunogenic to allow for a 1-dose regimen.

## 6 Adjuvanted rabies vaccines

Adjuvants enhance immune responses through so-called danger signals that drive maturation of immature dendritic cells into professional antigen presenting cells or by providing antigen depots that prolong antigen presentation. Alum, the most commonly used adjuvant, does not increase immune responses to the rabies vaccine [48]. Second generation adjuvants based on ligands for Toll-like receptors (TLR) seem to perform better [49]. One adjuvanted rabies vaccine called PIKA vaccine composed of Rabipur and a polyinosinic-polycytidylic acid based, TLR-3-activating adjuvant, has completed a phase II clinical trial [50]. The vaccine was given in an accelerated regimen with 2 doses on day 0, 2 doses on day 3 and one dose on day 7 and compared to Rabipur given at the regular day 0, 3, 7 and 14 regimen. The PIKA vaccine was well tolerated. Antibody titers ≥0.5 IU/ml increased faster than with the non-adjuvanted vaccine. Overall the trial showed comparative efficacy of the PIKA vaccine to the currently used rabies vaccine. Larger scale trials are needed to assess if the adjuvanted vaccine reliably accelerates onset of protective titers of rabies virus neutralizing antibodies, which would be highly advantageous for PEP. Other adjuvants have been tested pre-clinically in combination with the rabies vaccine and in general results indicated that they allowed for dose-sparing [51], [52], [53].

## 7 Genetically modified rabies virus

Rabies virus can be modified by genetic engineering [54]. Genes such as those encoding the phosphoprotein [55] or the matrix protein [56] can be deleted which attenuates the virus. Rabies viruses with such deletions do not replicate in animals including those with immunodeficiencies and are thus presumably safe. Matrix protein-deleted rabies virus is more immunogenic than virus with a phosphoprotein deletion or inactivated wild-type virus [57]. In spite of such promising immunogenicity data, the modified viruses grow less vigorously in cell culture than wild-type virus, which will likely raise production issues and increase vaccine cost. Also, public acceptance of a live rabies vaccine might be limited. Alongside using deletion mutants, vaccine viruses carrying two or three glycoprotein gene copies have also been developed [58]. In murine studies, inactivated virus showed increased immunogenicity compared to wild-type virus. Such viruses, provided their growth allows for scaled production, could be explored for PrEP.

## 8 Genetic vaccines

Genetic vaccines can be divided into RNA vaccines, DNA vaccines and viral vector vaccines. They have in common that a gene rather than a protein antigen is transferred. In case of RNA or DNA vaccines the gene only encodes for a rabies virus antigen, which results in a highly focused immune response unlike viral vectors, which induce T and B cell responses to antigens of the gene carrier as well. Uptake of different types of genetic vaccines depends on the vaccine carrier. Viral vectors commonly bind to specific cell surface receptors and are therefore readily internalized. Nevertheless, pre-existing neutralizing antibodies to viral vectors induced by previous natural infections can severely dampen their uptake and immune responses to a transgene product [59], [60]. Similarly, a viral vector should not be given repeatedly as vector neutralizing antibodies induced by the first immunization will impact the effectiveness of subsequent boosts. This is not a concern for RNA or DNA vaccines. Transfer of RNA or DNA vaccines across cell membranes is rather inefficient but can be facilitated by various physical or chemical methods [61].

## 9 RNA vaccines

A clinical phase I trial with an RNA rabies vaccine expressing the rabies virus glycoprotein, termed CV7201, tested a three-dose regimen in healthy adult volunteers. The vaccine was well tolerated but protective titers of antibodies were not achieved in all vaccinated individuals, titers declined by one year after vaccination and upon an additional boost failed to increase in all participants [62]. The RNA vaccine was thus clearly inferior to commercially available vaccines.

## 10 DNA vaccines

Multiple pre-clinical trials have shown that DNA vaccines given to mice [63], [64], [65] or monkeys [66], [67], [68] protect against rabies virus in PrEP or even PEP regimens. DNA vaccines against diseases other than rabies have been tested in multiple phase I and II trials with varied success. On phase IIb studies that tested a prime boost regimen composed of a DNA vaccine prime followed by an adenovirus vector boost for prevention of HIV-1 infection failed to meet its endpoint, and was stopped prematurely [69]. Another phase III trial for a CMV vaccine by Astellas also failed (ClinicalTrials.gov Identifier: NCT01877655). An additional phase III trial following promising phase II results [70] has been initiated with a therapeutic DNA vaccine for oncogenic types of human papillomavirus-associated tumors. The vaccine is delivered by electroporation and it should be pointed out that it aims to induce T cell responses rather than a rabies DNA vaccine that needs to target B cells.

Thus far overall available clinical data show that DNA vaccines are poorly immunogenic in humans. This may in part be overcome by improved delivery methods such as electroporation. Whether the use of such devices is practical in developing countries remains to be explored.

## 11 Viral vector vaccines

The safest and most immunogenic of the different types of viral vector vaccines are based on E1-deleted adenoviruses [71]. Adenovirus vectors are safe for the E1 deletion renders them replication-defective. There are several potential reasons why they are exceptionally immunogenic compared to other viral vectors. The E1-deletion reduces transcription of the viral antigens without affecting expression of the transgene product, which is typically under the control of a potent ubiquitously active promoter. This focuses the immune response largely on the transgene product rather than on antigens of the vaccine carrier. Adenovirus vectors are not cytolytic and they persist at low levels in a transcriptionally active form for an extended period of time thus constantly providing internal boost to the immune system. Immune responses to adenovirus vectors thus remain stable over long periods of time [72]. Non-human serotypes have been vectored [73], which avoids potential dampening of vaccine efficacy by pre-existing neutralizing antibodies to the vaccine carrier [74]. In non-human primates adenovirus vectors expressing the rabies virus glycoprotein were shown to induce long-lasting protective titers of neutralizing antibodies after a single dose [35], [75]. As adenoviruses persist albeit a low doses in a transcriptionally active form antibody titers are sustained [72]. Methods for storage without need for a cold-chain have been developed facilitating the use of such vaccines in resource-poor countries [76]. It has been estimated that upon mass production the cost of a single dose adenovirus rabies vaccine would be significantly lower than that of a 3-dose course of a traditional rabies vaccines [77]. This in turn would allow for their use in childhood immunization programs in highly endemic regions.

Other recombinant viral vectors have been explored. They were either too reactogenic, lacked efficacy or required prime-boost regimens. Furthermore, most them have not yet undergone clinical testing as vaccine carriers and it is thus unclear if methods for scale-up, purification and release testing that meet regulatory requirements can be achieved. Poxvirus vectors have been tested extensively in experimental animals [78], [79], [80], [81]. They are less immunogenic than adenoviral vectors [82], which may in part relate to a dampening of immune responses to the transgene product by potent immune responses to the numerous antigens of the vaccine carrier. Poxvirus vectors based on vaccinia virus are also reactogenic [83], while those based on more attenuated poxviruses such as Modified Vaccinia Ankara lack efficacy [81]. A rabies vaccine based on parainfluenza virus showed efficacy in mice if given twice in a prime-boost regimen; single dose regimens were not explored [84]. Long-lasting protection of dogs was observed upon a single immunization with a high dose of a Newcastle Disease Virus (NDV) rabies vaccine [85]. NDV is highly pathogenic for birds and it is transmissible to humans; potential toxicity of a recombinant NDV in either species needs to be explored further. A single dose of an attenuated pseudorabies virus expressing the rabies virus glycoprotein induced neutralizing antibodies in dogs [86]. The study did not assess protection against challenge.

## 12 Summary

Next generation human rabies vaccines are needed to reduce cost and numbers of doses for PrEP and PEP. It could be advantageous to develop different vaccine platforms for these two applications as each has their unique requirement for optimally protective immune responses. For PrEP vaccines that induce long-lived rabies virus neutralizing antibody and sustained memory B cell responses after a single dose would be needed and replication-defective adenoviral vectors may fulfill these requirements. There is currently no human rabies vaccine platform on the horizon that has been shown pre-clinically in a relevant animal model such as non-human primates to accelerate antibody production upon PEP and allow for a reduction in vaccine doses and potentially RIG. Further tools are still required to prevent rabies replication following the onset of clinical disease.

For review of rabies virus, epidemiology, diagnosis, prevention, and management: consult Fooks et al. in Nature Reviews Disease Primers [87].

## Conflict of Interest Statement

None declared.
